# CD31‐labeled circulating endothelial cells as predictor in anlotinib‐treated non‐small‐cell lung cancer: Analysis on ALTER‐0303 study

**DOI:** 10.1002/cam4.1584

**Published:** 2018-06-01

**Authors:** Zhujun Liu, Jing Wang, Zhaoting Meng, Xinyue Wang, Cuicui Zhang, Tingting Qin, Jinliang Chen, Xiangli Jiang, Liuchun Wang, Li Lin, Xiaoling Zhang, Peng Chen, Chun Huang, Richeng Jiang, Kai Li

**Affiliations:** ^1^ National Clinical Research Center for Cancer Tianjin Medical University Cancer Institute and Hospital Tianjin China; ^2^ Key Laboratory of Cancer Prevention and Therapy Tianjin China; ^3^ Tianjin’s Clinical Research Center for Cancer Tianjin China; ^4^ Department of Thoracic Oncology Tianjin Lung Cancer Center Tianjin Cancer Institute & Hospital Tianjin Medical University Tianjin China

**Keywords:** angiogenesis, anlotinib, CD105, CD31, endothelial cells, non‐small‐cell lung cancer

## Abstract

Our previous studies revealed that the level of activated circulating endothelial cells (aCECs) was correlated with the progression‐free survival (PFS) in antiangiogenesis therapy. Anlotinib displayed affirmatory efficacies in several clinical trials of non‐small‐cell lung cancer (NSCLC). To find a marker predicting the efficacy of anlotinib treatment, we investigated the correlations of aCECs with PFS and overall survival (OS) in patients with NSCLC treated with anlotinib and the impact of anlotinib on human umbilical vascular endothelial cells (HUVECs). The blood samples of 78 patients with NSCLC were collected. aCECs were identified by flow cytometry as CD45^−^/CD146^+^/CD31^+^ cells and CD45^−^/CD146^+^/CD105^+^ cells. The mean value of baseline aCECs counts was defined as the cutoff value, according to which patients were divided into high and low baseline groups. Statistical correlation between high baseline CD31‐labeled aCECs counts and number of metastatic lesions (>3) (χ^2 ^= 4.905, *P* = .027) was analyzed. The 49 patients treated with anlotinib were stratified according to the ratio of minimal aCECs counts at any time points to baseline (aCECs min/baseline) as <1 or ≥1. Interestingly, the patients with aCECs (CD31) min/baseline <1 displayed longer PFS [HR = 0.439, 95%CI (0.211‐0.912), *P* = .023]. The biological effect of anlotinib on HUVECs was investigated using MTT assays. Western blot analysis was conducted to evaluate the expression levels of CD31 and CD105 under anlotinib treatment and the underlying mechanisms. In vitro experiment data demonstrated that CD31 exhibited more sensitive changes than CD105 under anlotinib treatment through PI3K‐AKT pathway. Thus, our finding provides new insights into the mechanism by which the CD31‐labeled aCECs are a more sensitive marker for predicting the efficiency of anlotinib treatment.

## 1 INTRODUCTION

Circulating endothelial cells (CECs) are usually known as markers that indicate the status of new micrangium when vessels are injured and regrew. Moreover, the CEC level is significantly higher in patients suffered cancer than in healthy volunteers,^1^, ^2^, ^3^ suggesting that CECs are related to angiogenesis induced by the malignant tumor. Total CECs consist of endothelial progenitor cells (EPCs) mobilized from the bone marrow by tumor angiogenesis factors and the mature CECs derived and differentiated from EPC^4^, ^5^ or shed from the wall of the micrangium.^6^ Early EPCs express CD34^+^/CD133^+^/VEGFR‐2^+^, and late EPCs express CD133^−^/VEGFR‐2^+^/CD105^+^/CD62E^+^/CD31^+^/CD146^+^/CD144^+^/VWF^+^. Accompanied by cell differentiation, CD133 gradually decreases, while CD62E, CD31, CD105, and CD146 emerge. Importantly, only late EPCs or mature activated CECs (aCECs) can exert main vascular formation process.^7^ CECs have been indicated as a potential biomarker to reflect the extent of cancerous angiogenesis in a variety of malignancies. Furthermore, our previous studies revealed the level of aCECs may have the correlation with progression‐free survival (PFS) in antiangiogenesis therapy.^8^, ^9^

Anlotinib is a multitarget receptor tyrosine kinase inhibitor which inhibits vascular endothelial growth factor receptor (VEGFR) 1‐3, fibroblast growth factor receptor (FGFR) 1‐4, platelet‐derived growth factor receptors (PDGFR) α/β, c‐Kit, and Met, that is, has a broad spectrum of inhibitory action on angiogenesis and malignancies growth. In addition, anlotinib showed antitumor activity on tumor cells carrying mutations in epidermal growth factor receptor (EGFR).^10^, ^11^ Phase I clinical trial has established the safety profile of anlotinib and identified commended dose of 12 mg once daily at the 2 weeks on treatment followed by 1 week off treatment schedule.^11^ In phase II, as a third‐line and above treatment for patients with advanced non‐small‐cell lung cancer (NSCLC), anlotinib has got an affirmatory efficacy on patients with advanced NSCLC, in ALTER0302 double‐blind, controlled trial.^12^

In order to identify peripheral markers for predicting therapeutic efficacy, we conducted a tracking observation on aCECs levels in the ALTER‐0303 study to investigate the correlation between aCECs, PFS, and OS of anlotinib treatment.

Moreover, to confirm the effect of anlotinib on vascular endothelial cells and to find ideal markers representing such effect and elucidate its molecular basis, we examined the experiment of anlotinib treatment on human umbilical vascular endothelial cells (HUVECs) and investigated the mechanism of anlotinib’s function.

## 2 MATERIALS AND METHODS

### 2.1 Eligibility and exclusion criteria

This study was conducted in Tianjin Medical University Cancer Institute and Hospital from February 2015 to July 2016. All 78 patients were documented as advanced NSCLC.

Eligible patients included patients between the ages of 18‐75 years. Performance status of patients was evaluated using Eastern Cooperative Oncology Group (ECOG) performance status score. Patients with ECOG performance status 0‐1 and an expectation of longer than 3 months’ survival time are eligible in this study. Patients were pathologically diagnosed as stage III and IV of NSCLC and with at least one measurable lesion. Before entering the group, tumor tissues (tissue specimen or malignant pleural effusion) were tested of EGFR mutation and anaplastic lymphoma kinase (ALK) rearrangement by fluorescence in situ hybridization detection or sequencing, respectively. Both EGFR mutation‐positive patients and ALK fusion gene rearrangement patients needed to have documented disease progression or unacceptable adverse reactions after EGFR‐tyrosine kinase inhibitors (EGFR‐TKIs) therapy or ALK‐TKIs therapy, respectively, and those patients were also needed to have disease progression after at least one line of chemotherapy. Patients without EGFR mutation or ALK rearrangement needed to have disease progression after at least 2 lines of chemotherapy. The patients in this study are also required of adequate main organ function: hemoglobin (Hb) ≥ 90 g/L; absolute neutrophil count (ANC) ≥1.5 × 10^9^/L; platelet (PLT) ≥80 × 10^9^/L; total bilirubin (TBIL) ≤ 1.5 × upper limit of normal (ULN); alanine aminotransferase (ALT) and aspartate aminotransferase (AST) ≤ 2.5 × ULN, with liver metastasis ≤5 × ULN; serum creatinine (Cr) ≤1.5 × ULN or creatinine clearance rate (CCR) ≥60 mL/min; and left ventricular ejection fraction (LVEF) ≥low limit of normal (50%).

Exclusion standard included patients who had been used anlotinib, patients with small cell lung cancer (including small cell mixed with NSCLC), patients who had EGFR mutation or ALK rearrangement had not accepted related targeted treatment, patients who had central squamous lung cancer and squamous lung cancer with cavity or NSCLC with hemoptysis (>50 mL/d), patients who had other malignancies within the last 5 years, patients who had used chemotherapy drugs or received major surgery, cut biopsy, or obviously traumatic injury within the last 4 weeks, brain metastasis patients with symptoms or symptoms control time <2 months, patients with hemorrhagic tendency or had history of bleeding within the last 4 weeks or had arteriovenous thrombosis event within the last 6 months.

The study was conducted according to the principles of the Declaration of Helsinki (as revised in Fortaleza, Brazil, October 2013) and Good Clinical Practice requirements. The Ethical Committee approval number was E2015006 approved by Tianjin Medical University Institute and Cancer Hospital.

### 2.2 Therapy schedule

Patients were randomized into the anlotinib or the placebo capsule arms in 2:1 by central distribution table. In the anlotinib group, anlotinib was administrated 12 mg qd orally continuously for 2 weeks and stopped for 1 week, which was 3 weeks for 1 cycle. In the placebo group, placebo capsule was also distributed daily orally similarly. The treatment procedure in both groups was performed every 3 weeks until the patients met the criteria for progressive disease, experienced unacceptable toxicity, or patients’ withdrew of consent. Patients were stratified based on sex, age, tumor pathological type, disease stage, smoking history, No. of metastasis lesions, EGFR mutation, and ECOG performance.

### 2.3 Blood collection

Blood samples were collected before treatment (baseline) and then on the 7th, 15th, 21st, 42nd, and 63rd day of anlotinib or placebo. All blood samples were anticoagulated with EDTA and stored at 4°C before use. All the blood samples were tested within 9 hours from collection. Flow cytometry (FCM) was used to identify aCECs (CD45^−^/CD146^+^/CD31^+^ and CD45^−^/CD146^+^/CD105^+^). CD146 (342014), CD31 (303116), and CD105 (323204) were purchased from BioLegend (San Diego, CA), CD45 (IM3548U) was from Beckman Coulter (Marseille Cedex, France). Whole anticoagulated peripheral blood (100 μL) was added in the isotype control tube and stained with 10 μL of PC7, PerCP/Cy5.5, APC, and FITC IgG1 isotype control antibodies for 20 minutes. The same procedure was performed in the test tube incubated for 20 minutes with 10 μL of CD45‐PC7, CD146‐PerCP/Cy5.5, CD31‐APC, and CD105‐FITC antibodies, respectively. After incubation, red blood cells were lysed with lysing solution (Beckman Coulter) for 30 minutes. Afterward, cells were rinsed twice and resuspended in shell liquid. Using FS/SS gating strategy, acquisition was performed by FCM (Beckman Coulter, EPICS‐XL) equipped with a 488‐nm argon‐ion laser. A minimum of 100 000 events per sample was analyzed.

Data from each sample were analyzed by Software‐System II (Beckman Coulter). aCECs were identified using a sequential gating strategy. All results were showed by examination of CD45^−^/CD146^+^/CD31^+^ and CD45^−^/CD146^+^/CD105^+^ cells as a percentage, respectively.

### 2.4 Evaluation of efficacy

CT or MRI examinations were performed pre‐ and post‐therapeutically one cycle and every two cycles later or at any time during therapy, if necessary. Efficacies were evaluated according to NCI‐proposed Response Evaluation Criteria In Solid Tumors (RECIST1.1). Physical and imaging examination of tumor lesions should be carried out at least 4 weeks after the first effect evaluation in patients with complete remission, partial remission, and stable disease to confirm efficacy excepting progressive disease.

Progression‐free survival and overall survival (OS) were also documented. PFS is considered as the time from randomization to tumor progression or death from any reason. OS is the moment from the beginning of randomization to the cause of death for any reason.

### 2.5 Cell culture

HUVECs were purchased from the Shanghai Institute of Biochemistry and Cell Biology, Chinese Academy of Sciences (Shanghai, China). All cells were maintained in DMEM medium (Gibco, USA) supplementary with 10% fetal bovine serum (FBS) (Gibco, USA) and 100 units/mL of penicillin and streptomycin (Gibco, USA). Cells were incubated at 37°C in a humidified atmosphere of 5% CO_2_.

### 2.6 Reagents

Antibodies for CD31 (ab28364) and CD105 (ab11414) were purchased from Abcam (Cambridge, London, UK). Antibodies for p‐AKT (4060), AKT (4685), and GAPDH (5174) were purchased from Cell Signaling Technology (Danvers, MA, USA). HRP‐conjugated secondary antibodies were from Cell Signaling Technology (Danvers, MA, USA).

### 2.7 Cell proliferation assay

The 3000 cells per well were cultured in 96‐well plates for 24 hours and then were treated with Anlotinib for 24 hours. The MTT assay (Solarbio, Beijing, China) was used to evaluate cell viability upon cells according to the manufacturer’s protocol. Cell viabilities were calculated by measuring the optical density at 490 nm, using a spectrophotometric plate reader (BioTek, USA). All cell viability results were tested by three independent experiments.

### 2.8 Protein isolation and Western blot

Total protein was extracted from homogenized cells in RIPA buffer and subjected to 10% SDS‐PAGE. The proteins were transferred onto polyvinylidene difluoride membranes (Roche Molecular Biochemicals, Quebec, Canada), blocked with 5% BSA for 1 hour at RT, and then immunoblotted overnight at 4°C with appropriate primary antibodies against target proteins. The blots were further incubated with HRP‐conjugated secondary antibodies and developed with the ECL System (Millipore, Billerica, MA).

### 2.9 Statistical analysis

The two‐sided test was used for all statistical tests. *P* < .05 is considered as statistical significance. The mean ± SD or median (min, max) for statistical description was used in measurement data. Chi‐square and Fisher’s exact tests (accurate probabilistic method) were used to analyze the efficacy of placebo and anlotinib group. The Kaplan‐Meier test and Cox regression model were used to assess the survival difference between the two stratifications.

According to the mean value of baseline aCECs counts, 78 patients were divided into high and low groups. High and low group’s patients’ baseline aCECs levels were studied with clinical characteristics using chi‐square test. The correlations between the ratio of the minimum to baseline count values of aCECs and PFS, OS were analyzed using the Kaplan‐Meier method and Cox regression model.

In vitro experiment data were subjected to variance analysis (ANOVA), unpaired Student’s *t* test.

## 3 RESULTS

Seventy eight patients were enrolled in this study. Characteristics of the population are summarized in Table 1. No statistical difference was found between the characteristics of each group (all *P* > .05).

**Table 1:** Baseline characteristics of patients

Characteristics	Placebo (n = 26)	Anlotinib (n = 52)
No. of patients (%)		
Age (y)		
Median (range)	59.4 (44‐68)	58.9 (44‐74)
<60	11 (42.3)	26 (50)
≥60	15 (57.7)	26 (50)
Gender		
Male	18 (69.2)	32 (61.5)
Female	8 (30.8)	20 (38.5)
Smoking history		
Never	7 (26.9)	21 (40.4)
Ever	17 (65.4)	28 (53.8)
Still	2 (7.7)	3 (5.8)
Pathology		
Adenocarcinoma	22 (84.6)	44 (84.6)
Squamous or adenosquamous carcinoma	4 (15.4)	5 (9.6)
Other subtypes or undistinguishable	0	3 (5.8)
Clinical stage		
IIIB	2 (7.7)	2 (3.8)
IV	24 (92.3)	50 (96.2)
No. of metastatic lesions		
≤3	16 (61.5)	29 (55.8)
>3	10 (38.5)	23 (44.2)
EGFR mutation		
Yes	11 (42.3)	11 (21.2)
No	15 (57.7)	41 (78.8)
ECOG performance		
0	1 (3.8)	9 (17.3)
1	24 (92.3)	42 (80.8)
2	1 (3.8)	1 (1.9)

ECOG, Eastern Cooperative Oncology Group.

### 3.1 Response to therapy

The efficacy of 4 patients in the anlotinib and 8 in the placebo group was not determined. The disease control rate (DCR) was significantly benefited from anlotinib treatment (*P* < .0001). Out of 52 patients of anlotinib treatment, the median administration duration was 7.83 cycles (23.49 weeks) (Table 2).

**Table 2:** The response to treatment

Response Rate, n (%)	Placebo, n = 26	Anlotinib, n = 52
PR	1 (3.85)	3 (5.77)
SD	10 (38.46)	41 (78.85)
PD	7 (26.92)	4 (7.69)
NE	8 (3.08)	4 (7.69)
ORR	1 (3.85)	3 (5.77)
DCR^a^	11 (42.31)	44 (84.6)

PR, partial remission; SD, stable disease; PD, progression disease; NE, no evaluation; ORR, objective response rate; DCR, disease control rate.

a *P* < .0001.

### 3.2 PFS and OS

**Figure 1:**
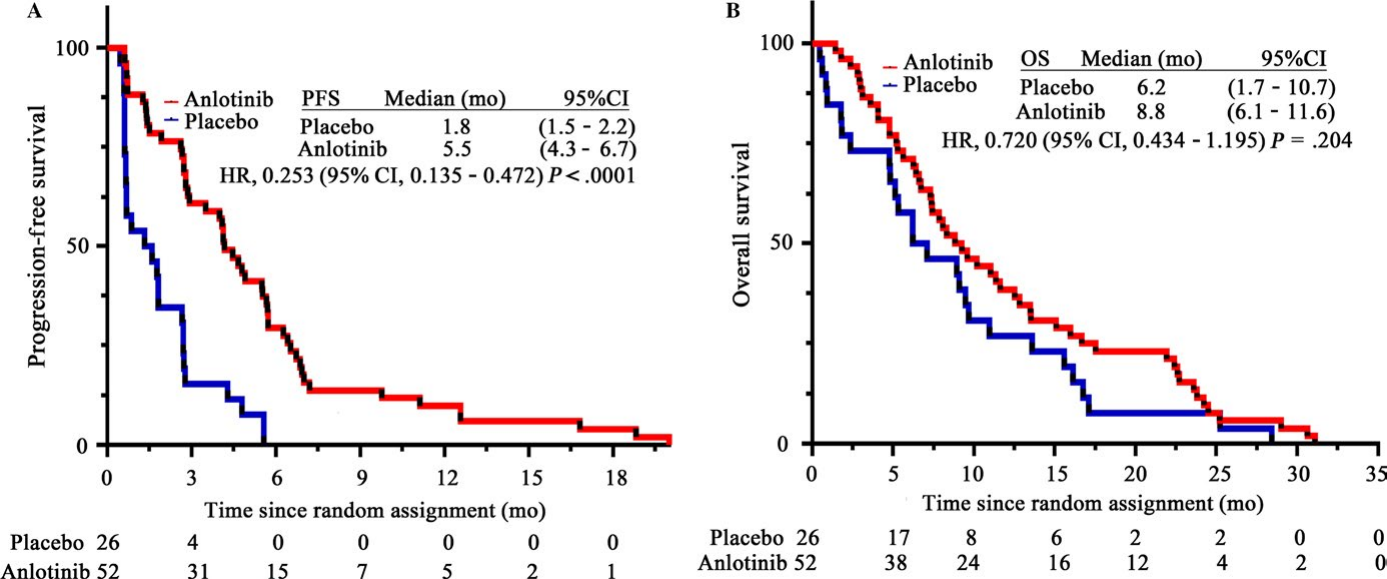
Kaplan‐Meier and Cox regression model analysis of PFS and OS between placebo and anlotinib group. A, PFS of the two groups. B, OS of the two groups. HR, hazard ratio; PFS, progression‐free survival; OS, overall survival

Patients were followed up in 20.14 months (range 10.43‐31.63 months) until October 2017. A significant difference in PFS was found between placebo and anlotinib group (χ^2^ = 21.420, *P* < .0001), while there was no statistical difference in OS (χ^2 ^= 1.631, *P* = .204) (Figure 1A,B).

### 3.3 Baseline aCECs level

**Figure 2:**
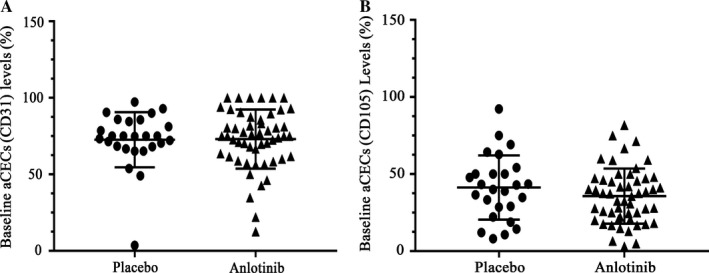
Baseline aCECs levels of placebo group and anlotinib group. A, CD31‐labeled aCECs levels of the two groups. B, CD105‐labeled aCECs levels of the two groups. aCECs, activated circulating endothelial cells

There was no significant difference between baseline aCECs levels before treatment in placebo group and anlotinib group (*P* > .05) (CD31‐labeled aCECs, Figure 2A; CD105‐labeled, Figure 2B).

### 3.4 Baseline aCECs counts and characteristics

There were no statistically correlations of aCECs baseline counts with patients’ baseline characteristics including age, gender, smoking history, ECOG performance, histology, stage, or EGFR mutations both in anlotinib and placebo group (*P* > .05). According to the mean value of baseline aCECs counts (72.93%), 78 patients were divided into high and low baseline groups. High baseline aCECs (CD31) counts were statistically related to the number of metastatic lesions (>3) (χ^2 ^= 4.550, *P* = .033) (Table 3).

**Table 3:** Baseline aCECs (CD31) level with the number of metastatic lesions

Number of Metastatic Lesions	High Baseline Group	Low Baseline Group
>3	30	15
≤3	14	19
Total	44	34

### 3.5 aCECs levels and survival

**Figure 3:**
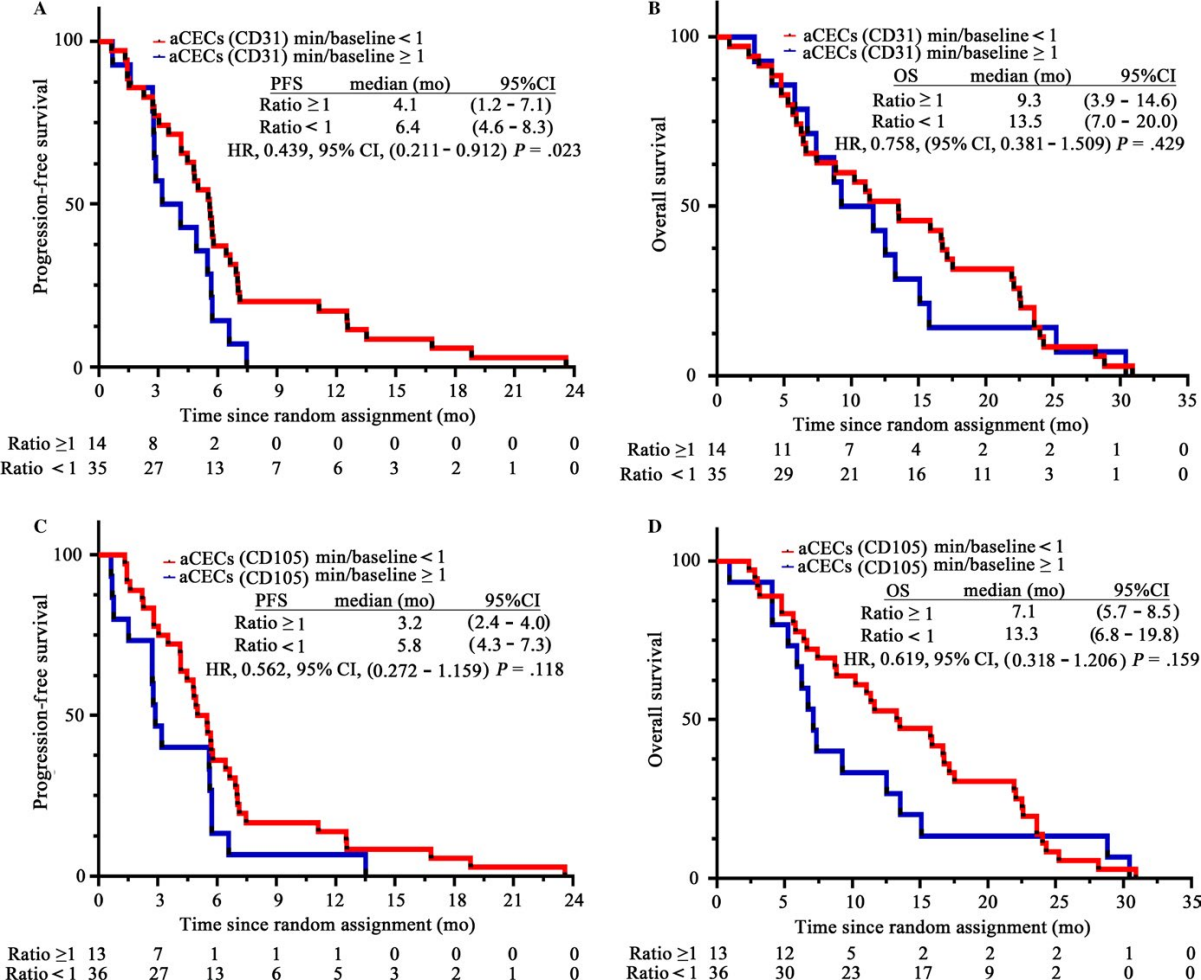
Analysis of PFS and OS in anlotinib group with a cutoff value of aCECs min/baseline = 1. A, PFS of CD31‐labeled aCECs min/baseline <1 and aCECs min/baseline ≥1 group. B, OS of the two groups. C, PFS of CD105‐labeled aCECs min/baseline <1 and aCECs min/baseline ≥1 group. D, OS of the two groups. PFS, progression‐free survival; OS, overall survival. aCECs, activated circulating endothelial cells

The 49 out of 52 patients with anlotinib treatment were stratified according to the ratio of minimum aCECs counts at each time points to baseline (aCECs min/baseline) as <1 or ≥1. In univariate survival analysis, patients with CD31‐labeled aCECs min/baseline <1 displayed longer PFS [HR = 0.439, 95%CI (0.211‐0.912), *P* = .023] (Figure 3A). However, there was no statistical association of OS with CD31‐labeled aCECs min/baseline ratio (Figure 3B). CD105‐labeled aCECs had no statistical association with PFS or OS (Figure 3C,D). There was no correlation between aCECs min/baseline ratio and PFS (or OS) in placebo arm.

### 3.6 Anlotinib inhibits HUVECs’ proliferation

**Figure 4:**
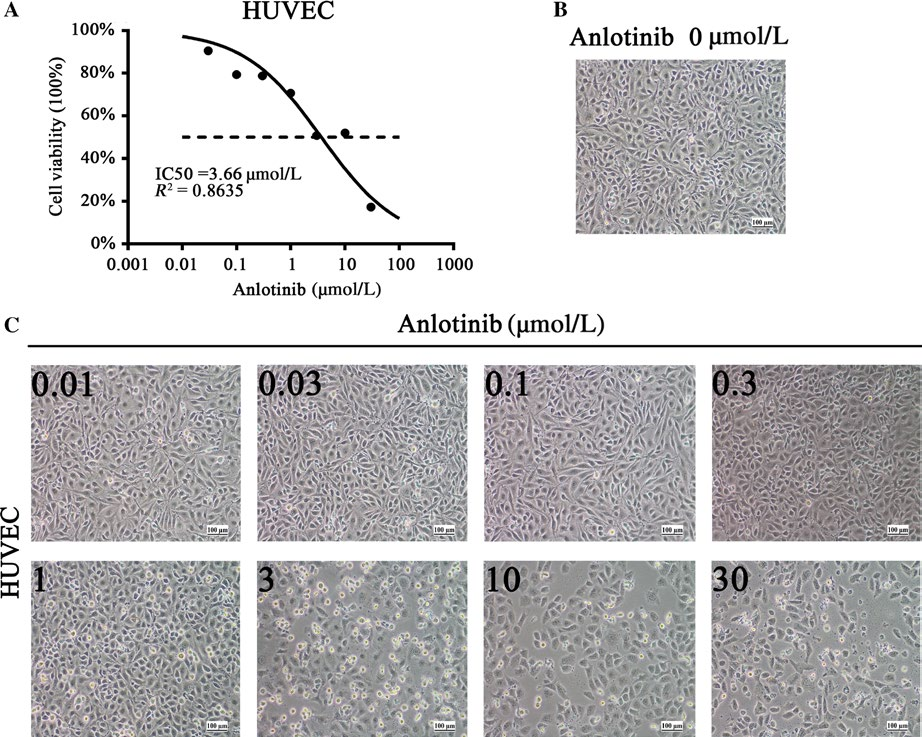
Anlotinib inhibits HUVEC cells’ proliferation. A, IC50 of anlotinib on HUVEC cells. B, Representative image of HUVEC cells without anlotinib treatment. C, Typical images of HUVEC cells with increasing concentration of anlotinib treatment for 24 hours in 6‐cm dishes. Magnification, x100.These experiments were repeated three times. HUVEC, human umbilical vascular endothelial cell

HUVEC cells were planted in 96‐well plates, treated with different concentrations of anlotinib (0.01‐100 μmol/L) for 24 hours. The IC50 of anlotinib treatment on HUVEC cells is 3.66 μmol/L (Figure 4A). We found that HUVEC cells obviously died under the treatment of 3 μmol/L anlotinib (Figure 4C).

### 3.7 CD31 is more sensitive to represent anlotinib’s impact on HUVECs than CD105

**Figure 5:**
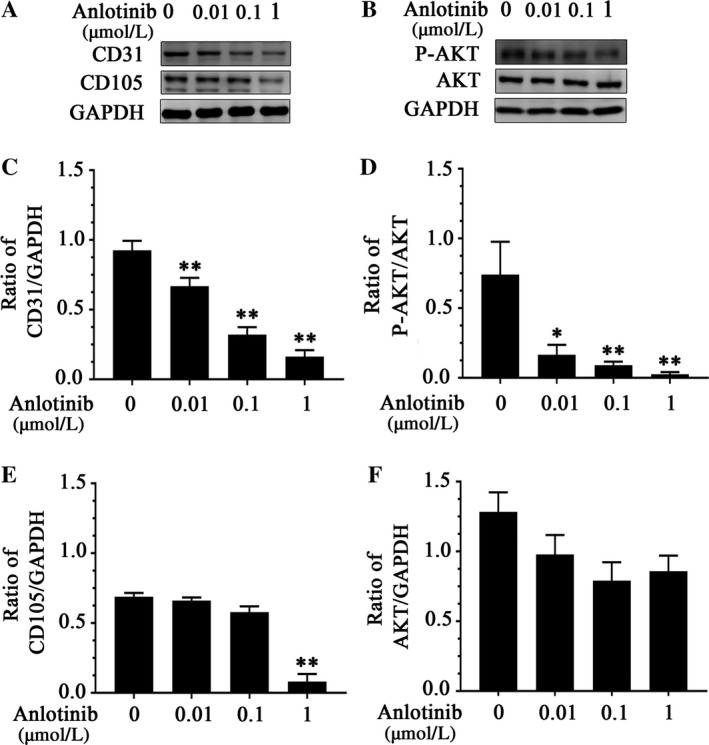
CD31 responds more sensitive to anlotinib treatment than CD105 in HUVEC cells via PI3K‐AKT pathway. A, CD31 protein was down‐regulated by Western blot analysis following the anlotinib treatment at various concentrations. CD105 protein was down‐regulated at the concentration of 1 μmol/L. B, Changes in P‐AKT protein levels following anlotinib treatment. C, Quantitative analyses of CD31 protein levels with anlotinib treatment. Values are normalized to GAPDH. D, Quantitative analyses of P‐AKT protein levels with anlotinib treatment. Values are normalized to AKT. E, Quantitative analyses of CD105 protein levels with anlotinib treatment. Values are normalized to GAPDH. F, Quantitative analyses of AKT protein levels with anlotinib treatment. Values are normalized to GAPDH. * *P *<* *.05, **P <* *.01.These experiments were repeated three times. The gray value of the western bands was measured by ImageJ software three times. HUVEC, human umbilical vascular endothelial cell

To determine the effect of anlotinib on CD31 and CD105 in HUVEC, CD31 and CD105 expression were measured. HUVECs were treated with anlotinib in 0.01, 0.1, and 1 μmol/L. A considerable decrease in CD31 protein levels with 0.01 μmol/L was determined by Western blot analysis (Figure 5A,C), and the CD105 protein levels were decreased in the concentration of 1 μmol/L (Figure 5A,E).

### 3.8 PI3K‐AKT signaling pathway is involved in downregulation of CD31 induced by anlotinib in HUVECs

To find out whether PI3K‐AKT signaling pathway is involved in the downregulation of CD31 induced by anlotinib, we treated the cells with 0.01, 0.1, 1 μmol/L anlotinib, respectively. Anlotinib treatment remarkably led to the reduction in P‐AKT at the protein levels (Figure 5B,D), indicating that PI3K‐AKT signaling is responsible for the anlotinib‐mediated downregulation of CD31 in HUVEC cells.

## 4 DISCUSSION

Non‐small‐cell lung cancer is believed as the leading cause of cancer‐related death worldwide. Although response rates to cytotoxic chemotherapy for NSCLC are approximately 30%‐40%, patients only have a median survival of 8‐10 months due to the resistance to therapy.^13^ Therefore, further understanding of the biology of NSCLC has led to the emergence of angiogenesis inhibition therapy. Angiogenesis is a complex process involving in sustaining malignant microenvironment, tumor growth, and metastasis.^14^ VEGF family, PDGF, EGF, and fibroblast growth factor (FGF) play important roles in angiogenesis, in which VEGFA (VEGF) is the most important initiator of angiogenesis.^15^

Bevacizumab is the first humanized monoclonal antiangiogenesis agent directly against VEGF. Clinical trial ECOG4599 demonstrated that Bevacizumab significantly prolonged the PFS and OS of patients with nonsquamous NSCLC due to its reinforcing and maintaining chemotherapeutic efficacy.^16^, ^17^ But several later clinical trials such as AVAPERL, POINTBREAK, and AVAIL declared that bevacizumab only prolonged the PFS rather than OS.^18^, ^19^, ^20^ Furthermore, it has been reported that bevacizumab enhances the proliferation and invasiveness of glioblastoma cells.^21^ Above mentioned evidence suggests that ‘improper’ single‐targeted therapy on VEGF pathway can simultaneously activate other pathways. Thereby, aroused cancer cell malignant behavior leads to failure of antiangiogenic therapy and shorter OS, so that it is urgent to identify ideal biomarkers to early predict efficacy and alarm progressive disease.

Unfortunately, up to now, there are no any validated markers to predict tumor’s response before antiangiogenesis therapy. Main reason for that includes at least two difficulties, one is the uncertainty of malignant cells’ development trend after antiangiogenic therapy, which comprises ‘dormant status’^22^ due to starvation in blood supply and activated stem‐like cell transformation induced by antiangiogenic hypoxia.^23^ For above reason, it’s hard to predict antiangiogenic efficacy aheading treatment through the expression extent of angiogenesis‐related factors on endothelial cells of micrangium around cancer, like what we did on EGFR mutation on cancer cells for predicting EGFR‐TKIs’ efficacy. Instead, we have to choose observation in the dynamic change in angiogenesis‐related factors, especially in blood, during therapy. However, another difficulty is the complexity of angiogenesis‐related factors which are composed of at least several dozens of determinants scarcely to be wholly measured. As the target of bevacizumab, VEGF has firstly been thought as candidate on the efficacy prediction for a long time, but its predictive effect has also been constantly controversial. A clinical trial based on bevacizumab plus docetaxel on breast cancer showed no correlations between serum VEGF level and PFS were found.^24^ However, another clinical study of bevacizumab with docetaxel on metastatic breast cancer showed high serum VEGF levels anticipated a good prognosis for bevacizumab.^25^ VEGF was also observed in the ECOG4599, AVAiL, AVF2107, AVOREN, and AVF2938trials, but results showed that baseline VEGF levels cannot predict bevacizumab‐based regimens’ efficacy.^26^ In the phase I, clinical trials of rh‐endostatin, VEGF, and b‐FGF level in blood did not reveal any regular fluctuations during therapy, not to mention it was impossible to use them to predict efficacy or prognosis.^27^ The reason for above failures may be ascribed to the fact that angiogenesis is a complex process in which a dynamic balance between the antagonized effect of angiogenesis inducers and inhibitors exists every time so VEGF’s effect should be assuaged by many angiogenesis inhibitors, and it is hard to know the whole angiogenetic status only through VEGF level change. Therefore, people transfer their attention to some more downstream effectors that should be closer to the formation of the vasculature and less subject to the interference by other upstream factors, in order to get more accurate prediction. Among them, aCECs have been put forward as a promising biomarker for prognosis of NSCLC.^1^, ^28^, ^29^, ^30^ Our previous studies have also demonstrated that aCECs (CD105) is a powerful predictive marker in chemotherapy with rh‐endostatin in NSCLC, so aCECs was chosen in this study again.

In recent years, the research on CECs has been more frequent, but there are still no recognized markers to define CECs. In this study, we selected the widely used markers such as CD45, CD146, CD133, CD105, and CD31. CD45 is an indispensable marker for the removal of CD45^+^ lymphocytes. CD146 is an essential marker as it can distinguish CECs from CD45^−^ blood cell populations. CD133 is a marker of early EPC, which gradually becomes negative during cell proliferation and differentiation to mature EPC to be able to form vasculature.^7^ In our study, we defined aCECs as active mature cells that are able to form new blood vessels, so we did not choose CD133 as a label. CD105 is preferentially expressed on the vascular endothelium of tumor tissue,^31^ and it is largely confined to tumor margin that is the active angiogenesis region. Hewett and Murray^32^ found that CD31 expressed on endothelial cells, so it can be used as a comprehensive marker of endothelial cells in the tissue. Studies have shown that compared with the pan‐endothelial cell marker (CD31), CD105 accurately reflects the proliferation status of endothelial cells.^33^, ^34^, ^35^ We believe that CD31 is expressed on all vessels in tumor tissue, while CD105 is predominantly expressed on newly formed blood vessels that are mainly on the frontier infiltration of the tumor. So far, there are no data to prove which label (CD31 or CD105) of CECs is better for prediction of prognosis. Studies showed that CD105‐labeled CECs was a predictive marker of longer PFS or/and OS,^1^, ^28^, ^29^ while other study found that the reduction in CD31‐labeled CECs was associated with longer survival.^30^ Therefore, in our study, we chose CD45^−^/CD146^+^/CD105^+^ and CD45^−^/CD146^+^/CD31^+^ as two measuring classifications of aCECs.

Our results revealed that the min/baseline of CD31‐labeled aCECs in 63 days (3 cycles) below 1 was associated with longer PFS (6.4 months vs 4.1 months, *P* = .023), as to OS, there was no statistical difference between aCECs min/baseline ratio in 63 days (3 cycles) <1 or ≥1 (*P* = .429). While CD105‐labeled aCECs has no statistical correlation with PFS and OS even though there was a tendency of a meaningful *P* value (*P* = .118 and *P* = .159). This result highlights that CD31‐labeled aCECs are more sensitive than CD105 in predicting the efficacy of anlotinib treatment. Our previous studies revealed that CD105‐labeled aCECs count difference was related to time to progression under treatment of rh‐endostatin,^8^, ^9^ which was different from our data. The reason for the difference is probably the different antiangiogenic agent used in this study. Actually, both CD31 and CD105 have been defined as markers of active circulating endothelial cells, we made single and bilabeling in this study and found that CD31 was an ideal marker for aCECs.

Anlotinib is a multitarget receptor tyrosine kinase inhibitor which suppresses neoplastic angiogenesis and tumor growth. According to the RECIST 1.1 criteria, in phase II study, the anlotinib group has got objective response rate (ORR) of 10% (*P* = .028), DCR of 83.3% (*P* < .0001), and median PFS of 4.83 months (95%CI, 3.47‐6.40) (*P* < .0001), which was statistically superior to the placebo group.^12^ Our study showed that the anlotinib group got ORR of 5.77% (*P* > .05), DCR of 84.6% (*P* < .0001), and median PFS of 5.50 months (95%CI, 4.30‐6.70) (*P* < .0001) compared with the placebo group in 78 cases in our site, well in accordance with ones in total 437 cases (data not shown). As to OS, there were no statistical differences between anlotinib and placebo group (8.8 vs 6.2 months) in our study, which is different from the data in total cases in all sites (9.6 vs 6.3 months) probably due to the small pool of cases in our site.

Different from typical antiangiogenesis drugs such as rh‐endostatin and bevacizumab, anlotinib inhibits both neoplastic angiogenesis and tumor growth pathways.^10^, ^11^ Actually, it has been reported that activation of the EGFR pathway increases the production of tumor‐secreted VEGF that acts on endothelial cells to promote angiogenesis.^36^ Obviously, single‐targeted antiangiogenic drugs, such as bevacizumab and rh‐endostatin, cannot restrain tumor cells’ subsequent activated effect on VEGF by EGFR so they may only decrease ‘most active fraction’ instead of the entirety of CECs within long‐term therapy. To contrast, anlotinib is a powerful inhibitor of VEGFR/VEGF, Met, and EGFR, which is able to induce apoptosis of CECs and suppress production of VEGF induced by EGFR pathway in cancer cells. Moreover, anlotinib is an inhibitor of c‐kit that is a marker of EPCs and an important role in stem cell maintenance and differentiation,^37^ so that it made a rapid decline of both CD31‐ and CD105‐labeled CECs. Given that CD31‐labeled CECs are in the large majority, the reduction was obvious enough to reveal meaningful statistical result, while CD105‐labeled aCECs were only in a small population of CECs, which might be interfered easier by many factors. Our data also indicated that anlotinib downregulated CD31 on concentration of 0.01 μmol/L, while downregulated CD105 on concentration of 1 μmol/L. It suggests that CD31‐labeled aCECs may be a more sensitive marker as a PFS predictor when it comes to multitargeted and ‘two domains’ (endothelial cells and neoplastic cells) antiangiogenic medicine, even during initial short‐term detection. Furthermore, we found that the high CD31‐labeled aCECs level was associated with the number of metastasis lesions (>3), which demonstrated that the counts of aCECs were in proportional to the tumor burden.

In conclusion, we highlight that CD31‐labeled aCECs are a sensitive marker of predictive value of the efficacy of anlotinib treatment. The decline of these cells in a short‐term detection (63 days, 3 therapeutic cycles) indicates benefit in PFS under anlotinib treatment. Anlotinib‐induced CD31 downregulation is likely the result of probably through PI3K‐AKT signaling pathway activation. It would be valuable to lead further studies in this direction in order to confirm the molecular basis and clinical reliability of CD31‐labeled aCECs in the prediction of antiangiogenic therapeutic efficacy.

## TRIAL REGISTRATION

This work included two registered clinical trials: 1. To Predict Efficacy by Detecting Circulating Endothelial Cell Subsets and Blood Perfusion Parameters Changes in Vivo Tumor in the Phase II/III Study of Anlotinib in Patients With Advanced Non‐small‐cell Lung Cancer; NCT02029209; 2. ALTER0303; NCT02388919.

## ETHICAL STATEMENT

All patients were fully informed and had signed informed consent.

## CONFLICT OF INTEREST

Anlotinib used in in vitro experiments was donated by Nanjing Chia Tai TianQing Company. All authors had full access to all of the data in the study and had final responsibility for the decision to submit for publication. All authors have no conflict of interest.

## References

[1] Kawaishi M , Fujiwara Y , Fukui T , et al. Circulating endothelial cells in non‐small cell lung cancer patients treated with carboplatin and paclitaxel. J Thorac Oncol. 2009;4:208‐213. 19179898 10.1097/JTO.0b013e318193030d

[2] Mehran R , Nilsson M , Khajavi M , et al. Tumor endothelial markers define novel subsets of cancer‐specific circulating endothelial cells associated with antitumor efficacy. Cancer Res. 2014;74:2731‐2741. 24626092 10.1158/0008-5472.CAN-13-2044 PMC4024326

[3] Najjar F , Alammar M , Bachour M , Al‐Massarani G . Circulating endothelial cells as a biomarker in non‐small cell lung cancer patients: correlation with clinical outcome. Int J Biol Markers. 2014;29:e337‐e344. 25041783 10.5301/jbm.5000100

[4] Beaudry P , Force J , Naumov GN , et al. Differential effects of vascular endothelial growth factor receptor‐2 inhibitor ZD6474 on circulating endothelial progenitors and mature circulating endothelial cells: implications for use as a surrogate marker of antiangiogenic activity. Clin Cancer Res. 2005;11:3514‐3522. 15867254 10.1158/1078-0432.CCR-04-2271

[5] Furstenberger G , von Moos R , Lucas R , et al. Circulating endothelial cells and angiogenic serum factors during neoadjuvant chemotherapy of primary breast cancer. Br J Cancer. 2006;94:524‐531. 16450002 10.1038/sj.bjc.6602952 PMC2361171

[6] Beerepoot LV , Mehra N , Vermaat JS , Zonnenberg BA , Gebbink MF , Voest EE . Increased levels of viable circulating endothelial cells are an indicator of progressive disease in cancer patients. Ann Oncol. 2004;15:139‐145. 14679134 10.1093/annonc/mdh017

[7] Duda DG , Cohen KS , di Tomaso E , et al. Differential CD146 expression on circulating versus tissue endothelial cells in rectal cancer patients: implications for circulating endothelial and progenitor cells as biomarkers for antiangiogenic therapy. J Clin Oncol. 2006;24:1449‐1453. 16549839 10.1200/JCO.2005.04.2861 PMC2718681

[8] Liu ZJ , Wang J , Wei XY , et al. Predictive value of circulating endothelial cells for efficacy of chemotherapy with Rh‐endostatin in non‐small cell lung cancer. J Cancer Res Clin Oncol. 2012;138:927‐937. 22331237 10.1007/s00432-012-1167-5 PMC11824260

[9] Wang J , Xiao J , Wei X , et al. Circulating endothelial cells and tumor blood volume as predictors in lung cancer. Cancer Sci. 2013;104:445‐452. 23298271 10.1111/cas.12097 PMC7657163

[10] Lin B , Song X , Yang D , Bai D , Yao Y , Lu N . Anlotinib inhibits angiogenesis via suppressing the activation of VEGFR2, PDGFRbeta and FGFR1. Gene. 2018;654:77‐86. 29454091 10.1016/j.gene.2018.02.026

[11] Sun Y , Niu W , Du F , et al. Safety, pharmacokinetics, and antitumor properties of anlotinib, an oral multi‐target tyrosine kinase inhibitor, in patients with advanced refractory solid tumors. J Hematol Oncol. 2016;9:105. 27716285 10.1186/s13045-016-0332-8 PMC5051080

[12] Han B , Li K , Zhao Y , et al. Anlotinib as a third‐line therapy in patients with refractory advanced non‐small‐cell lung cancer: a multicentre, randomised phase II trial (ALTER0302). Br J Cancer. 2018;118:654‐661. 29438373 10.1038/bjc.2017.478 PMC5846072

[13] Schiller JH , Harrington D , Belani CP , et al. Comparison of four chemotherapy regimens for advanced non–small‐cell lung cancer. N Engl J Med. 2002;346:92‐98. 11784875 10.1056/NEJMoa011954

[14] Folkman J . Tumor angiogenesis: therapeutic implications. N Engl J Med. 1971;285:1182‐1186. 4938153 10.1056/NEJM197111182852108

[15] Ferrara N . Molecular and biological properties of vascular endothelial growth factor. J Mol Med (Berl). 1999;77:527‐543. 10494799 10.1007/s001099900019

[16] Sandler A , Gray R , Perry MC , et al. Paclitaxel‐carboplatin alone or with bevacizumab for non‐small‐cell lung cancer. N Engl J Med. 2006;355:2542‐2550. 17167137 10.1056/NEJMoa061884

[17] Sandler A , Yi J , Dahlberg S , et al. Treatment outcomes by tumor histology in Eastern Cooperative Group Study E4599 of bevacizumab with paclitaxel/carboplatin for advanced non‐small cell lung cancer. J Thorac Oncol. 2010;5:1416‐1423. 20686429 10.1097/JTO.0b013e3181da36f4

[18] Barlesi F , Scherpereel A , Rittmeyer A , et al. Randomized phase III trial of maintenance bevacizumab with or without pemetrexed after first‐line induction with bevacizumab, cisplatin, and pemetrexed in advanced nonsquamous non‐small‐cell lung cancer: AVAPERL (MO22089). J Clin Oncol. 2013;31:3004‐3011. 23835708 10.1200/JCO.2012.42.3749

[19] Patel JD , Bonomi P , Socinski MA , et al. Treatment rationale and study design for the pointbreak study: a randomized, open‐label phase III study of pemetrexed/carboplatin/bevacizumab followed by maintenance pemetrexed/bevacizumab versus paclitaxel/carboplatin/bevacizumab followed by maintenance bevacizumab in patients with stage IIIB or IV nonsquamous non‐small‐cell lung cancer. Clin Lung Cancer. 2009;10:252‐256. 19632943 10.3816/CLC.2009.n.035

[20] Reck M , von Pawel J , Zatloukal P , et al. Phase III trial of cisplatin plus gemcitabine with either placebo or bevacizumab as first‐line therapy for nonsquamous non‐small‐cell lung cancer: AVAil. J Clin Oncol. 2009;27:1227‐1234. 19188680 10.1200/JCO.2007.14.5466

[21] Simon T , Coquerel B , Petit A , et al. Direct effect of bevacizumab on glioblastoma cell lines in vitro. Neuromolecular Med. 2014;16:752‐771. 25113866 10.1007/s12017-014-8324-8

[22] van der Veldt AA , Lubberink M , Bahce I , et al. Rapid decrease in delivery of chemotherapy to tumors after anti‐VEGF therapy: implications for scheduling of anti‐angiogenic drugs. Cancer Cell. 2012;21:82‐91. 22264790 10.1016/j.ccr.2011.11.023

[23] Xu Y , Li Q , Li XY , Yang QY , Xu WW , Liu GL . Short‐term anti‐vascular endothelial growth factor treatment elicits vasculogenic mimicry formation of tumors to accelerate metastasis. J Exp Clin Cancer Res. 2012;31:16. 22357313 10.1186/1756-9966-31-16 PMC3310846

[24] Miles D , Cameron D , Bondarenko I , et al. Bevacizumab plus paclitaxel versus placebo plus paclitaxel as first‐line therapy for HER2‐negative metastatic breast cancer (MERiDiAN): a double‐blind placebo‐controlled randomised phase III trial with prospective biomarker evaluation. Eur J Cancer. 2017;70:146‐155. 27817944 10.1016/j.ejca.2016.09.024

[25] Miles DW , de Haas SL , Dirix LY , et al. Biomarker results from the AVADO phase 3 trial of first‐line bevacizumab plus docetaxel for HER2‐negative metastatic breast cancer. Br J Cancer. 2013;108:1052‐1060. 23422754 10.1038/bjc.2013.69 PMC3619079

[26] Hegde PS , Jubb AM , Chen D , et al. Predictive impact of circulating vascular endothelial growth factor in four phase III trials evaluating bevacizumab. Clin Cancer Res. 2013;19:929‐937. 23169435 10.1158/1078-0432.CCR-12-2535

[27] Herbst RS , Hess KR , Tran HT , et al. Phase I study of recombinant human endostatin in patients with advanced solid tumors. J Clin Oncol. 2002;20:3792‐3803. 12228199 10.1200/JCO.2002.11.061

[28] Ilie M , Long E , Hofman V , et al. Clinical value of circulating endothelial cells and of soluble CD146 levels in patients undergoing surgery for non‐small cell lung cancer. Br J Cancer. 2014;110:1236‐1243. 24473396 10.1038/bjc.2014.11 PMC3950863

[29] Sanchez Hernandez A , Jose Juan O , Vidal Martinez J , et al. Quantification of circulating endothelial cells as a predictor of response to chemotherapy with platinum and pemetrexed in patients with advanced non‐squamous non‐small cell lung carcinoma. Clin Transl Oncol. 2015;17:281‐288. 25236392 10.1007/s12094-014-1223-5

[30] Yuan DM , Zhang Q , Lv YL , et al. Predictive and prognostic significance of circulating endothelial cells in advanced non‐small cell lung cancer patients. Tumour Biol. 2015;36:9031‐9037. 26084612 10.1007/s13277-015-3657-y

[31] Weidner N , Semple JP , Welch WR , Folkman J . Tumor angiogenesis and metastasis–correlation in invasive breast carcinoma. N Engl J Med. 1991;324:1‐8. 10.1056/NEJM199101033240101 1701519

[32] Hewett PW , Murray JC . Human microvessel endothelial cells: isolation, culture and characterization. In Vitro Cell Dev Biol Anim. 1993;29A:823‐830. 8167895 10.1007/BF02631356

[33] Akagi K , Ikeda Y , Sumiyoshi Y , et al. Estimation of angiogenesis with anti‐CD105 immunostaining in the process of colorectal cancer development. Surgery. 2002;131:S109‐S113. 11821796 10.1067/msy.2002.119361

[34] Fonsatti E , Jekunen AP , Kairemo KJ , et al. Endoglin is a suitable target for efficient imaging of solid tumors: in vivo evidence in a canine mammary carcinoma model. Clin Cancer Res. 2000;6:2037‐2043. 10815930

[35] Miyata Y , Sagara Y , Watanabe S , et al. CD105 is a more appropriate marker for evaluating angiogenesis in urothelial cancer of the upper urinary tract than CD31 or CD34. Virchows Arch. 2013;463:673‐679. 23975255 10.1007/s00428-013-1463-8 PMC3825622

[36] Larsen AK , Ouaret D , el Ouadrani K , Petitprez A . Targeting EGFR and VEGF(R) pathway cross‐talk in tumor survival and angiogenesis. Pharmacol Ther. 2011;131:80‐90. 21439312 10.1016/j.pharmthera.2011.03.012

[37] Russell JS , Brown JM . Circulating mouse Flk1 + /c‐Kit+/CD45‐ cells function as endothelial progenitors cells (EPCs) and stimulate the growth of human tumor xenografts. Mol Cancer. 2014;13:177. 25047738 10.1186/1476-4598-13-177 PMC4112847

